# Activated or Impaired: An Overview of DNA Repair in Neurodegenerative Diseases

**DOI:** 10.14336/AD.2021.1212

**Published:** 2022-07-11

**Authors:** Nan Qin, Anke Geng, Renhao Xue

**Affiliations:** Shanghai Key Laboratory of Maternal Fetal Medicine, Clinical and Translational Research Center of Shanghai First Maternity & Infant Hospital, Frontier Science Center for Stem Cell Research, School of Life Sciences and Technology, Tongji University, Shanghai, China

**Keywords:** DNA repair, neurodegenerative diseases, base excision repair, homologous recombination, nonhomologous end joining

## Abstract

As the population ages, age-related neurodegenerative diseases have become a major challenge in health science. Currently, the pathology of neurodegenerative diseases, such as Alzheimer's disease, Parkinson's disease, amyotrophic lateral sclerosis, and Huntington's disease, is still not fully understood. Remarkably, emerging evidence indicates a role of genomic DNA damage and repair in various neurodegenerative disorders. Here, we summarized the current understanding of the function of DNA damage repair, especially base excision repair and double strand break repair pathways, in a variety of neurodegenerative diseases. We concluded that exacerbation of DNA lesions is found in almost all types of neurodegenerative diseases, whereas the activities of different DNA repair pathways demonstrate distinct trends, depending on disease type and even brain region. Specifically, key enzymes involved in base excision repair are likely impaired in Alzheimer's disease and amyotrophic lateral sclerosis but activated in Parkinson's disease, while nonhomologous end joining is likely downregulated in most types of neurodegenerative diseases. Hence, impairment of nonhomologous end joining is likely a common etiology for most neurodegenerative diseases, while defects in base excision repair are likely involved in the pathology of Alzheimer's disease and amyotrophic lateral sclerosis but are Parkinson's disease, based on current findings. Although there are still discrepancies and further studies are required to completely elucidate the exact roles of DNA repair in neurodegeneration, the current studies summarized here provide crucial insights into the pathology of neurodegenerative diseases and may reveal novel drug targets for corresponding neurodegenerative diseases.

## 1. Introduction

Neurodegenerative diseases are progressive and functional disorders of the nervous system due to loss of neurons and active connections between cells, i.e., synapses. Most neurodegenerative diseases, such as Alzheimer's disease (AD), Parkinson's disease (PD), amyotrophic lateral sclerosis (ALS), and Huntington's disease (HD), are largely age-related. Currently, as the aging population continues to increase, the harmful effects of neurodegenerative diseases are also increasing. Moreover, current knowledge about the pathology of neurodegenerative diseases is still very limited, which makes drug discovery for neurodegenerative diseases a huge challenge. Further understanding the pathologies of these neurodegenerative diseases is a top priority of modern medical science.

The degeneration of the nervous system is a complex process that could be affected by a variety of environmental or genetic factors. It is currently believed that the major causes of neurodegeneration are oxidative stress, mitochondrial dysfunction, excitotoxicity, and immune inflammation [1-5]. Indeed, growing evidence also suggests the involvement of additional mechanisms. For example, more severe deoxyribonucleic acid (DNA) damage is found in the brains of neurodegenerative diseases [6]. The corresponding genome disruption might involve cell dysfunction and death.

**Figure 1.**
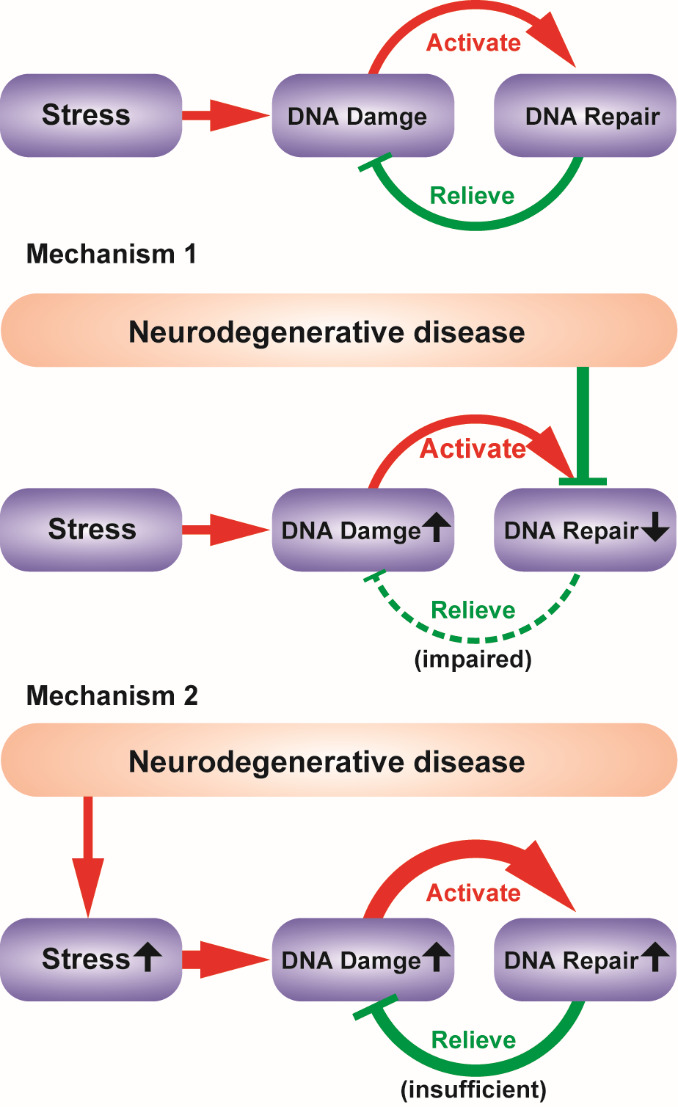
**Two possible mechanisms that may cause the increase of DNA damage in neurodegenerative diseases**. Stress induces DNA damage and DNA repair is activated to relieve damage. Neurodegeneration could either exacerbate DNA damage by suppressing DNA repair (DNA repair downregulated) or by enhancing stress (DNA repair upregulated).

The number of DNA lesions in the genome is mediated by the balance between damage and repair. Therefore, the exacerbation of genome fragmentation during neurodegeneration could result from either elevated damage or attenuated repair (Fig. 1). Theoretically, if enhanced stress induces extra DNA lesions, DNA repair mechanisms are expected to be activated. In contrast, if repair defects account for the increase in damage, an impairment of DNA repair activity is predicted to be observed. Hence, to determine the cause of the boost in DNA damage in various neurodegenerative disorders, it is necessary to determine whether the activity of DNA repair mechanisms is upregulated or downregulated. In this review, we will summarize current findings on the trends of activities of different DNA repair pathways in different neurodegenerative diseases. This will provide valuable clues for pathology studies and further illuminate the molecular mechanisms of neurodegenerative diseases.

## 2. DNA damage is exacerbated in neurodegenerative diseases

The integrity and stability of the genome are essential for cell survival and function. Genomic DNA (gDNA) plays a central role in organisms by encoding genetic information and consequently controlling all kinds of cellular processes. However, DNA is continuously subject to various types of destructive stresses from the environment, such as ultraviolet (UV) irradiation, ionizing radiation and carcinogenic chemical substances, and internal factors, such as reactive oxygen species (ROS) generated during metabolism and errors during DNA replication. These external and internal threads lead to DNA base damage, including base modification, mismatch, loss, cross-linking, conversion, etc., and DNA strand damage, including single strand breaks (SSBs) and double strand breaks (DSBs). The accumulation of DNA damage during aging causes genome instability, senescence and even cell death, resulting in functional loss and age-related disorders [7,8].

DNA damage also has a crucial impact on the nervous system. To the best of our knowledge, the majority of DNA damage in the brain is induced by oxidative stress. To drive signal transmission and processing in the intricate neuronal network, the brain consumes approximately 20% of the total energy, although it weighs only 2% of the total body. Such an extremely high metabolic rate leads to extensive oxidative stress in brain cells. Huge amounts of ROS are generated, which include superoxide anion (.O^2-^), hydroxyl radical (.OH), and hydrogen peroxide (H_2_O_2_). ROS usually play a destructive role by attacking biological macromolecules, including lipids, proteins, and DNA [9]. Due to oxidative stress, brain cells exhibit severe DNA oxidative damage [10]. Base oxidation is a common type of ROS-induced DNA lesion. The first identified and most frequently detected product of base oxidation is 8-hydroxyguanine (8-oxo-dG), which is often used as a marker of oxidation in DNA [11]. Interestingly, the abundance of 8-oxo-dG is enhanced in the brains of neurodegenerative diseases such as AD [12] and PD [13]. Clearly, DNA oxidative damage is associated with neurodegeneration.

Moreover, DSBs are much more toxic than base oxidation, as they may cause the loss of billions of bases. Cumulative oxidative damage or errors in replication can lead to DSBs [14]. In recent years, DSBs in the nervous system and neurodegenerative diseases have started to receive increasing attention. Usually, the formation of γH2AX foci, which is the phosphorylation of histone protein H2A variant X at Ser139, could be a marker of DSBs. A large increase in brain γH2AX foci was observed in AD [15] and PD [16] mouse models and AD patients [17]. Hence, DSBs, as well as oxidative damage, have been identified in neurodegenerative diseases, suggesting the potential involvement of DNA damage and genome fragmentation in neurodegeneration.

Notably, lesions in both nuclear DNA (mainly gDNA) and mitochondrial DNA (mtDNA) accumulate during aging and progressively lead to cell dysfunction and death in age-related diseases [18]. Although mtDNA has been more well studied in neurodegenerative diseases because of the close relationship between mitochondrial dysfunction and neurodegeneration [19,20], the role of nuclear DNA damage and repair should not be overlooked, as such damage influences the stability of the genome. Emerging evidence has demonstrated that nuclear DNA appears to be at least equally important as mtDNA in neurodegenerative diseases [21]. Therefore, this review will focus on the damage and repair of nuclear DNA.

## 3. DNA damage is repaired via several different pathways

To counteract injurious threats and maintain the stability and accuracy of the genome, a DNA repair system was established in living organisms during evolution. Regarding different classes of DNA damage, a few distinct repair pathways are present in all kinds of cells. Here, the mechanisms of different DNA repair pathways, namely, base excision repair (BER), nucleotide excision repair (NER), mismatch repair (MMR), and DSB repair, including homologous recombination (HR) and nonhomologous end joining (NHEJ), will be summarized.

### 3.1 Base excision repair (BER)

**Figure 2.**
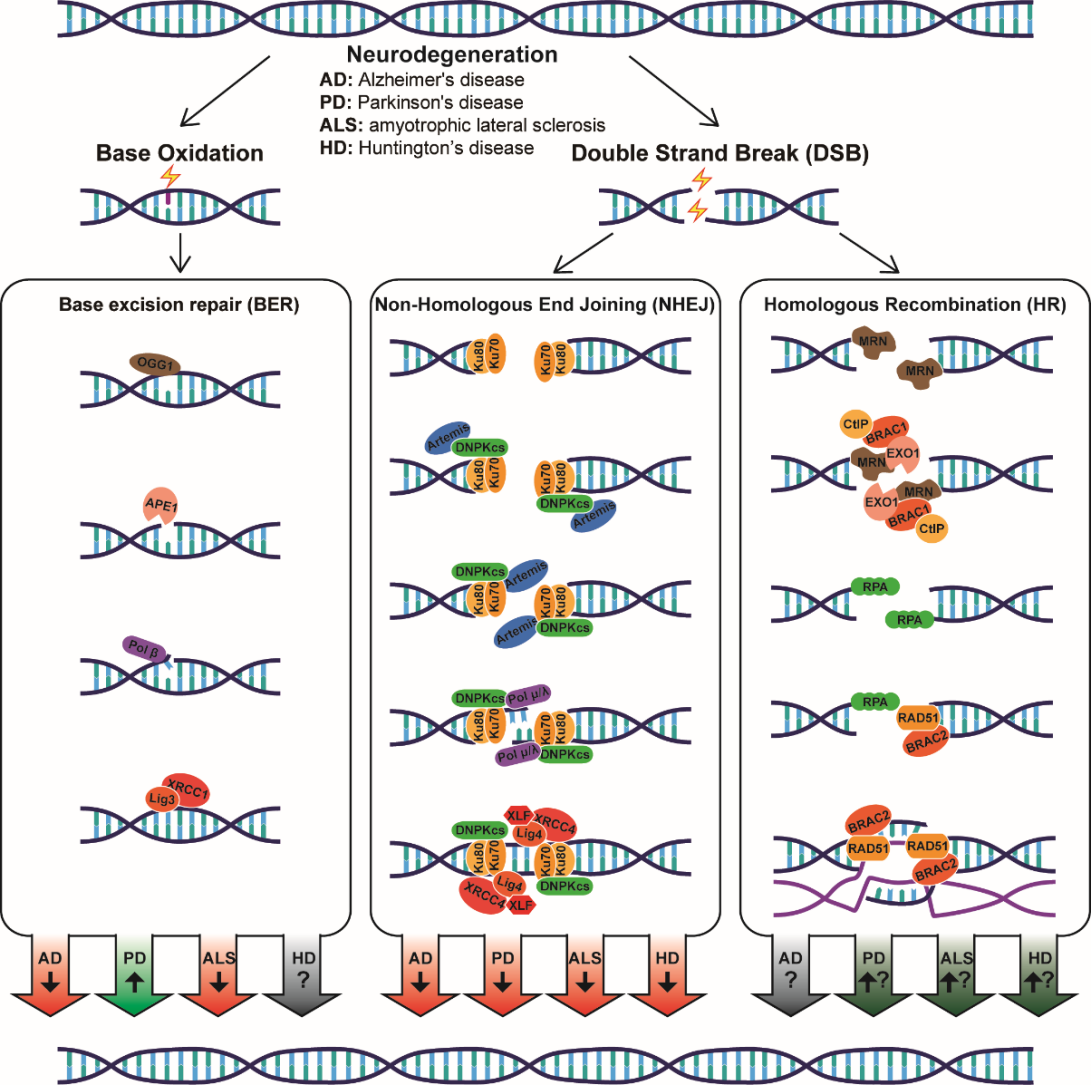
**During neurodegeneration, base oxidation and DSB are exacerbated, and the activity of different DNA repair pathways are altered**. According to current findings, it is likely that BER is downregulated in AD and ALS but upregulated in PD, NHEJ is downregulated in AD and ALS, while HR is upregulated in PD and ALS.

The BER pathway removes a large number of small and nonhelix-distorting base lesions, including base modification, i.e., alkylation, ring saturation and oxidation, and unexpected bases, i.e., uracil, thymine and hypoxanthine [22]. For example, 8-oxo-dG, a representative type of oxidative base as mentioned above, can be repaired via BER (Fig. 2). When the BER pathway is activated, damaged bases are identified and removed by DNA glycosylase. In mammals, there are 11 known DNA glycosylases, such as uracil DNA glycosylase (UDG), endonuclease VIII-like 1 (NEIL1), and 8-oxoguanine glycosylase 1 (OGG1). [23]. Among all these glycosylases, OGG1 is a typical enzyme that specifically removes 8-oxo-dG. Robust expression of OGG1 is also found in the nervous system, including the cerebellum, brainstem, and spinal cord, and the abundance and activity of OGG1 was found to increase with age in the mouse brain after 8 weeks [24]. Therefore, OGG1-dependent BER might be a key repair mechanism of DNA oxidative damage in the aging brain.

After base removing, an abasic (AP) site is left. Afterward, the AP sites are identified by purine/pyrimidine endonuclease (APE1). APE1 cleaves the AP site to form a nick, which is then patched by DNA polymerases. DNA polymerase β (Pol β) is specific for short patches of one single nucleotide, while polymerase ε (Pol ε) and polymerase δ (Pol δ) are responsible for long patches of 2-10 nucleotides. OGG1-induced 8-oxo-dG removal usually produces a nick of one single nucleotide that utilizes Pol β as a patcher. Finally, joining of the nucleotide chain is completed by a protein complex of XRCC1 and DNA ligase III (Lig3) [25,26].

In the nervous system, base oxidation is the most popular DNA lesion. As a result, the classic OGG1-APE1-Pol β-XRCC1-Lig3-mediated BER pathway, which specifically clears oxidative bases, is the most active DNA repair mechanism in the nervous system [24]. It is also highly likely that the activity of the BER pathway in the brain is age sensitive, as the expression levels of OGG1, APE1 and Lig3 were all found to change with age in a brain region-dependent manner [27,28]. Hence, BER is probably the most well-studied DNA repair pathway in the nervous system and neurodegenerative diseases. This topic will be introduced in detail later in this review.

### 3.2 Nucleotide excision repair (NER)

The NER pathway fixes a variety of bulk DNA lesions, such as helix distortion formed by UV-induced DNA 6-4 photoproducts and chemical-induced intrachain cross-linking [29]. There are two subtypes of the NER pathway: genome-wide NER (GG-NER), which can recognize and eliminate damage over the entire genome, and transcription-coupled NER (TC-NER), which mainly fixes DNA damage at the active transcript region [30].

First, DNA lesions are recognized by XPC-RAD23B dimers in GG-NER or by RNA polymerase in TC-NER [31]. Then, TFIIH, XPA, RPA, and XPG are recruited to the damage sites. The error nucleotides are cleaved by DNA endonucleases XPG and XPF/ERCC1 and removed by XPB/XPD. After that, DNA polymerase and proliferating cell nuclear antigen (PCNA) drive the synthesis of a new complementary sequence using the intact strand as a template. Finally, DNA ligase I (Lig1) seals the nick to complete the repair process [32].

### 3.3 Mismatch repair (MMR)

The MMR pathway corrects misincorporations of bases as well as insertions and deletions [33,34]. Initially, the mismatch site is recognized by MLH1. Afterward, the double strand of DNA is unfolded by helicase II, and the lesion area is excised by the DNA exonuclease EXO1. Such excision produces a gap in one single strand, which is consequently filled by DNA polymerase, and the incision is linked up by DNA ligase [35].

### 3.4 Double strand break (DSB) repair: homologous recombination (HR) and nonhomologous end joining (NHEJ)

Both HR and NHEJ are pathways for the repair of DSBs [36]. The HR pathway fixes DSBs with relatively high accuracy, as it uses homologous sister chromatids as templates. As a result, it is only activated in the S and G2 phases of the cell cycle [37]. During HR (Fig. 2), the MRN complex (MRE11/RAD50/NBS1), CtIP, BRAC1, EXO1 and BLM helicase orchestrate to remove the DNA ends and produce a new single-stranded 3’ DNA end, which is immediately wrapped by RPA to prevent the formation of a secondary structure and protects the DNA strand from nuclease digestion. Then, RAD51, with the assistance of BRCA2, replaces RPA and mediates chain exchange along with sister chromatids to finally complete homologous recombination repair [38].

Unlike HR, NHEJ does not require homologous chromosomes. Despite its relatively lower accuracy, NHEJ works independent of the cell cycle and acts as the main method of DSB repair in nondividing cells, i.e., neurons [39,40]. In NHEJ (Fig. 2), the Ku70/Ku80 heterodimer recognizes DSBs and subsequently initiates the autophosphorylation of DNA-PKcs. Then, DNA-PKcs and Artemis form a complex that functions as an endonuclease to remove DNA lesions. Polymerases μ (Pol μ) and λ (Pol λ) are two known DNA polymerases that synthesize new DNA chains to fix strand breaks during NHEJ. Finally, XLF, XRCC4 and DNA Ligase IV (Lig4) complete the reconstitution of double stranded DNA [41].

In addition, DSBs activate a critical kinase, ataxia telangiectasia mutated (ATM). Inherited mutations in ATM cause ataxia telangiectasia, which is a genetic nervous system disorder. In the case of DNA damage, ATM is activated by autophosphorylation at Ser1981, relocates to the damaged sites, phosphorylates a number of key DSB repair proteins, such as H2AX, p53, CHK2, 53BP1, NBS1, and BRCA1, and thereby further regulates the cell cycle state and determines the downstream repair pathways [42-44]. Moreover, ATM is also a protein with multiple functions. In response to oxidative stress, it can be directed stimulated by ROS in a DSB-independent manner [45] and play alternative roles other than DNA repair. For example, activated ATM functions as a redox sensor that contributes to antioxidative reactions [46], facilitates autophagy via the mTORC1 pathway [47,48], and maintains mitochondrial homeostasis [49]. Hence, the activity of ATM has been monitored in different neurodegenerative diseases, which will be introduced in detail below.

Both HR and NHEJ are essential DNA repair mechanisms in the nervous system. As DSBs usually occur early during the development of the nervous system, elimination of either the HR or NHEJ pathway leads to embryonic death [50]. In the adult and aged nervous systems, most neurons are well differentiated postmitotic cells. Therefore, the major pathway for DSB repair is NHEJ. On the other hand, HR mainly affects proliferating neural precursor cells, so it is more effective at the early embryonic stage. HR-deficient mice (XRCC2 KO) die at E9-10, much earlier than NHEJ-deficient mice (Lig4 KO) [50]. Hence, NHEJ has gained more attention than HR in the neurodegeneration of aged brains. Nonetheless, HR cannot be completely overlooked, as the mature nervous system also consists of large amounts of dividing cells, including neural progenitor cells and glial cells. Additionally, DNA damage might stimulate postmitotic neurons back into the cell cycle [51], and a special type of HR has also been detected in G0/G1 cells [52]. Regarding this, both NHEJ and HR will be discussed in this review.

## 4. DNA repair in neurodegenerative diseases

As described above, DNA repair mechanisms are present in the nervous system to rescue the destruction of DNA. Therefore, whether DNA repair plays a role in the pathology of neurodegenerative diseases has been a key question in the field of neuroscience for decades. As shown inFig. 1, one idea is that DNA repair pathways are blocked in the degenerative brain such that DNA oxidative lesions and strand breaks accumulate in the genome, resulting in cell dysfunction and death (Mechanism 1 inFig. 1). If this is true, DNA repair impairment could be considered one of the key etiologies of neurodegenerative diseases, as it directly causes the exacerbation of DNA damage. Another possibility exists that the intensified DNA lesions are not caused by lack of repair activity but by more severe oxidative stress (Mechanism 2 inFig. 1), which could result from enhanced ROS generation or impaired antioxidative activity. In this case, DNA repair pathway activity should be magnified by DNA injury rather than inhibited. However, the magnified repair activity is still insufficient to rescue the damage and stop the degeneration of nerve cells. If so, DNA repair may not be a primary etiology of neurodegenerative diseases.

To determine which mechanism (1 or 2) underlies neurodegeneration, it is necessary to determine whether DNA repair activity is upregulated or downregulated for each individual pathway in various neurodegenerative diseases. Attenuation of repair activity would support the former hypothesis that dysfunction of DNA repair is one of the primary causes of neurodegeneration. On the other hand, if the activity level rises, the latter hypothesis would be favored: DNA repair pathways do not directly involved in the pathogenesis of neurodegenerative diseases. Nevertheless, regarding the complexity of the nervous system and the DNA repair mechanism, a simple and straightforward answer may not be obtained. Indeed, repair activity may demonstrate completely distinct trends for different pathways, in different diseases, and in different regions of the brain. The aim of this review is to outline the tremendous studies concerning this question in the past few decades and try to reveal some clues to elucidate the true function of DNA repair during neurodegeneration.

### 4.1 Alzheimer's disease (AD)

AD is the most common neurodegenerative disease. Currently, nearly 50 million people suffer from AD or related dementia. Most AD patients are over 65 years old. The main clinical sign of AD is dementia, such as cognitive impairment and memory loss. The majority of AD is sporadic. Although the pathogenesis of AD is still not fully elucidated, studies have revealed that the AD brain exhibits β-amyloid peptide (Aβ) plaques, neurofibrillary tangles formed by the accumulation of hyperphosphorylated microtubule-associated protein tau, glial cell activation, neuroinflammation, and the loss of neurons and synapses [53]. Among them, the most well-determined hallmark of AD is Aβ plaques, which are aggregates of Aβ peptides, products of abnormal cleavage of amyloid precursor protein (APP) by β- and γ-secretase, in the hippocampus and neocortex. The most influential hypothesis regarding the pathology of AD is the amyloid cascade hypothesis [54]. This hypothesis suggests that deposition of Aβ leads to a series of cascade reactions, including the disruption of calcium homeostasis of neurons, neuronal energy metabolism impairment, oxidative stress induction, activation of microglia and astrocytes to produce inflammatory factors, and the loss of synapses and neuronal death. In this amyloid cascade, Aβ acts as the central initiation of the pathological process of AD. However, the amyloid cascade hypothesis is not sufficient to explain the complex mechanism of AD. For example, numerous drugs designed for Aβ cleanup failed in clinical trials, suggesting that alternative mechanisms other than Aβ might also be involved. Recently, DNA damage and repair have also been taken into consideration as putative etiologies of AD. A key question is whether DNA repair is inhibited or enhanced during AD.

DNA repair has been studied using tissues and cells from AD patients, animals, or cell model. As early as the 1980s, people began to study DNA damage and repair in AD using fibroblasts or lymphocytes from patients. These cells demonstrated higher sensitivity than normal cells when subjected to DNA disturbing external stimulations, such as chemicals [55,56], X-ray [57] and gamma irradiation [58]. Although the enhanced DNA damage response might be a result of diminished repair activity, no molecular biological assessment of repair pathways was carried out during this period. Moreover, no observations were made in neural tissue or cells at this stage.

Until the 1990s, studies using brain tissues from AD patients demonstrated enhanced oxidative DNA damage in the parietal, temporal, occipital, frontal lobe, superior temporal gyrus and hippocampus [59] and more severe DNA breaks in the cerebra-cortex [60]. Moreover, proteins involving different DNA repair pathways have been studied using biochemical and molecular biological approaches in various regions of the AD brain, which will be introduced in detail below.

Although some studies using AD cell models concerned the NER [61] and MMR [62] pathways, the majority of reported studies were focused on BER and DSB repairs, as base oxidation and DSBs are the most popular DNA lesions found in the nervous system. As described above, BER is the main mechanism to counteract base oxidation, such as 8-oxo-dG. OGG1 is a key glycosylase that recognizes 8-oxo-dG to initiate BER. In the brains of AD patients, whether the expression level of OGG1 is increased or decreased is still debated [63,64] and might be different in distinct brain regions [65]. Nonetheless, regardless of the expression level, the glycosylase activity of OGG1 was found to be impaired in almost all regions, including the hippocampal and parahippocampal gyri (HPG), superior and middle temporal gyri (SMTG), and inferior parietal lobule (IPL) [66], as well as the frontal (FL), temporal (TL), and parietal (PL) lobes of the cortex [63]. Indeed, the activity of OGG1 is likely modulated by posttranslational modification. For example, a recent study revealed that the loss of activity is likely caused by reduced deacetylation of OGG1 by histone deacetylase 1 (HDAC1) [67]. Additionally, polymorphisms may also affect the activity of OGG1. In AD patients, the Ser326Cys polymorphism in OGG1 led to more severe DNA damage [68,69], although it was not significantly associated with AD [70,71]. Moreover, the A53T and A288V polymorphisms of OGG1 were also identified in the AD brain. These two variants both exhibited significantly decreased catalytic activity [72]. In summary, the activity of OGG1 decreases in most regions of the AD brain. The reduction in activity could result from impaired deacetylation or polymorphisms. This activity deficiency could potentially cause the inhibition of BER.

During BER, APE1 is another key protein that cleaves AP sites. The expression of APE1 was found to decrease in a cell model using Aβ-treated human neuroblastoma cells [73] and an animal model using transgenic (Tg)-ArcSwe mice [64]. Nonetheless, in studies of post-mortem AD brain autopsies, increased expression of APE1 was found in the hippocampus [74] and cerebral cortex [75], decreased expression was observed in the entorhinal cortex [65], and no difference in the overall APE1 expression level was reported [76]. Taken together, the trend of APE1 expression in the AD brain is complicated and distinct in different regions. Moreover, in another study, CDK5-dependent phosphorylation of APE1 at Thr232 suppressed the activity of APE1 in the AD brain [77], suggesting that the activity of APE1 is diminished by abnormal protein modification in AD. This is consistent with the trend of OGG1. Thus far, it is likely that a rundown of BER activity is accompanied by the occurrence of AD in most brain regions. The elevation of APE1 expression in some regions could be explained as a compensation effect.

The next active enzyme in the BER pathway is Pol β, which fills new nucleotides into the gap of the DNA strand. The expression of Pol β was found to be attenuated in either the whole brain [76] or cerebellum [65] during AD. These observations are consistent with an impairment of BER. Interestingly, downregulation of Pol β in a heterozygous KO AD mouse model renders the mouse brain more vulnerable to neuronal injury [78], supporting the idea that Pol β-dependent BER contributes to the etiology of AD.

In the final step of BER, the DNA gap connection is completed by XRCC1 and Lig3. In patients with AD, the mRNA level of Lig3 was elevated in the frontal cortex but attenuated in the cerebellum [79]. In Aβ-treated human neuroblastoma cells, XRCC1 expression was impaired, but Lig3 expression was unchanged [73]. Apparently, the trend of protein expression was not uniform. Interestingly, two polymorphic variants of XRCC1 (Arg194Trp and Arg399Gln) were found to enhance DNA damage in AD patients [80]. The former (Arg194Trp) was shown to be associated with sporadic late-onset AD in Chinese patients [81], while the latter (Arg399Gln) had no significant association with AD [71]. As a result, the XRCC1 polymorphism might be a genetic inducer of BER dysfunction in AD. Again, reduced BER activity in AD can be speculated.

In summary, the expression level trend of key enzymes in the BER pathway is complex. However, there was an overall downward trend of protein activity (Table 1), including the downregulation of OGG1 and APE1 activity via protein modification, OGG1 and XRCC1 activity by polymorphisms, and Pol β activity by direct inhibition of expression. Hence, the activity of BER is likely impaired in the AD brain (Fig. 2).

Compared with BER, the repair of DSBs is even more important in the nervous system. A key kinase that activates a series of DSB repair proteins via phosphorylation is ATM. Mutation in ATM that eliminates kinase activity caused the death of neurons and glial cells and subsequently led to neurodegeneration similar to AD in Drosophila [82], suggesting a role of ATM in AD. However, no change in ATM expression was found in lymphocytes from AD patients [83]. The trend of ATM expression and activity in brain tissue is still not clear. More attention needs to be paid to ATM in future studies.

**Table 1:** The expression level and activity of a series of key enzymes for DNA damage repair are altered in neurodegenerative diseases.

	AD		PD		ALS		HD	
**OGG1**	expression decrease	[63]	expression increase	[112-114]	expression increase	[140]		
	expression increase	[64]						
	brain region dependent	[65]						
	activity decrease	[63,66,67]						
**APE1**	expression decrease	[64,65,73]	expression increase	[115]	expression decrease	[138-140]		
	expression increase	[74,75]			expression increase	[141]		
	expression no change	[76]			activity decrease	[138]		
	activity decrease	[77]						
**Pol β**	expression decrease	[65,76]	expression increase	[116]				
**Lig3**	brain region dependent	[79]						
	expression no change	[73]						
**XRCC4**	expression decrease	[73]	expression decrease	[117]				
**ATM**			activity increase	[16]	activity increase	[144-147]	expression increase	[164]
							activity increase	[163-165]
**BRAC1**	expression increase	[89]	expression increase	[124]	expression increase	[146]		
	expression decrease	[90]	activity increase	[126]				
**DNA-PKcs**	expression decrease	[84,86]						
	activity decrease	[84]						
**Ku80**					expression increase	[149]		
PARP1	expression increase	[92]	expression increase	[124]	expression increase	[147,148]		
	brain region dependent	[65]						
	activity increase	[92]						

As described above, HR and NHEJ are two different pathways for DSB repair, and NHEJ is more important than HR for adult and aged brains. In NHEJ, DSB first activates a key kinase, DNA-PKcs, which phosphorylates a series of substrates to initialize the downstream pathway. It was reported that DNA-PKcs was suppressed in the AD brain. Both the expression level and kinase activity were reduced in the cortex of the AD brain [84] and Aβ-treated PC12 cells [85]. The downregulation of DNA-PKcs was found in either neurons or astrocytes [86]. Interestingly, loss of DNA-PKcs makes cultured hippocampal neurons more vulnerable to Aβ-induced injury [87]. The deficiency of DNA-PKcs suggested a reduction of NHEJ in the AD brain.

BRCA1 is another important mediator of the repair of DSBs, especially in HR. Immunohistochemistry studies revealed that BRCA1 colocalized with phosphorylated tau in neurofibrillary tangles in AD brain autopsies, suggesting the potential involvement of BRCA1 in AD [88]. However, there are still discrepancies about the changes in BRCA1 expression in the AD brain. Reports of both increasing [89] and decreasing [90] expression levels can be found. Hence, further work is required to address the role of BRCA1 in AD, and the trend of HR activity in AD is still unclear.

Notably, PARP1 is a protein that plays key roles in several different types of DNA repair, including BER, HR and NHEJ. The function of PARP1 depends on poly-ADP-ribosylation of its substrates. In lymphocytes from AD patients, the expression level of PARP1 increases [91]. In the AD brain, the poly-ADP-ribosylation activity of PARP1, as well as the abundance of PARP1 protein, increases in the frontal and temporal lobes of the cortex [92]. Furthermore, a recent study also found that the expression of PARP1 is upregulated in the AD entorhinal cortex and hippocampus [65]. The only exception was that the abundance of PARP1 decreased in the cerebellum [65]. Hence, in most brain regions of AD patients, PARP1 is upregulated. In contrast, PARP1 was found to be downregulated in multiple cellular AD models. In a nerve cell line expressing α4β2-nicotinic receptors (nAChRs), a specific receptor of Aβ, less expression of PARP1 was found compared with control cells [93]. In PC12 cells expressing double Swedish mutation forms of human APP, the activity of PARP1 was impaired [94]. Such disagreement between cellular models and patients suggests that upregulation of PARP is more likely an inherent characteristic of AD rather than a secondary effect evoked by toxic Aβ treatment. Interestingly, the intrinsic elevation of PARP1 activity is not sufficient to eliminate DNA damage in the AD nervous system. A possible explanation is that final DNA repair activity is limited by blockage of pathways downstream of PARP1. In addition, it is likely that the boost of PARP1 activity might lead to cell death via parthanatos rather than protect cells from genome fragmentation.

Taken together, in the AD brain, the activity of NHEJ is likely suppressed due to downregulated DNA-PKcs. However, the process of HR is still elusive (Fig. 2 andTable 1). Based on current findings, it can be speculated that the overall repair capacity decreases in the AD brain, suggesting that defects in DNA repair could be a primary cause of AD.

### 4.2 Parkinson's disease (PD)

PD is the second most common neurodegenerative disease after AD and occurs mainly in the elderly population. The prevalence rate of PD in people over 60 years old is as high as 1% [95]. PD patients suffer from motor retardation and tremor due to loss of dopaminergic neurons in the substantia nigra of the midbrain and subsequent deficiency of dopamine (DA) in the striatum. Like AD, most PD cases are sporadic. The loss of dopaminergic neurons mainly results from oxidative stress, abnormal protein aggregates known as Lewy bodies, and mitochondrial dysfunction [96].

The main component of Lewy bodies is misfolded and aggregated α-synuclein [97]. α-synuclein is a 140 kDA protein that exists in the form of monomers and tetramers under physiological conditions, whereas oligomers and fibrils are its pathogenic forms found in PD [98]. The oligomers or fibrils of α-synuclein attack the cell membrane through the formation of transmembrane pores [99], alter mitochondrial membrane potential, disrupt the cytoskeleton [100], impair protein turnover [101,102], and induce inflammation [103]. Mitochondrial membrane depolarization or high levels of oxidative stress lead to mitochondrial injury. Physiologically, damaged mitochondria undergo mitochondrial phagocytosis and degradation, known as mitophagy. To drive mitophagy, PINK1 and Parkin are required for the ubiquitination of mitochondrial membrane proteins. Parkin is an E3 ubiquitin ligase that is activated by PINK1 via phosphorylation at Ser65 [103-105]. Dysfunctional mutations in PINK1 and Parkin were found in PD patients [106]. In addition, glutamatergic neurotoxicity, endoplasmic reticulum (ER) stress, etc., may also involve pathogenic mechanisms of PD.

Emerging evidence has also shown that DNA damage in both the nucleus and mitochondria is another important cause of neuronal injury in PD. Higher oxidative damage of DNA was observed in the brain than in the age-matched control brain [107,108]. For example, in a PD mouse model established using virus-delivered α-synuclein, a significant increase in the DSB marker γH2AX foci was found in dopaminergic neurons [16].

The presence of oxidative damage to DNA, such as 8-oxo-dG, in the PD brain indicates the importance of BER in PD [109]. Transgenic mice with knockout of OGG1, the key DNA glycosylase in BER, exhibited a PD-like phenotype, including severe depletion of striatal dopamine [110] and loss of tyrosine hydroxylase (TH)-positive neurons in the substantia nigra [111]. OGG1 knockout mice were also found to be more sensitive to a dopaminergic toxin 1-methyl-4-phenyl-1,2,3,6-tetrahydropyridine (MPTP) [111]. These studies confirmed that OGG1 plays a protective role in PD. Nevertheless, a trend of increased OGG1 expression in PD was found. The abundance of OGG1 in the substantia nigra is increased in the brains of PD patients [112]. Consistently, in PC12 cells, treatment with toxins that induce PD-like injury, such as melanin, MnCl_2_ or 1-methyl-4-phenylpyridinium (MPP^+^), elevated the expression level of OGG1 [113,114]. Unfortunately, the trend of OGG1 activity is still unclear. However, the current findings suggest that the OGG1-dependent BER pathway is likely activated to play a protective role in the response to PD injury, which is distinct from AD.

The expression level of APE1, the enzyme that removes AP sites in BER, was elevated in rotenone-treated MN9D dopaminergic neurons as a PD cell model [115]. Pol β, the functional DNA polymerase in BER, was upregulated in SH-SY5Y cells and substantia nigra neurons following exposure to rotenone as a PD model [116]. These trends are also different from AD. Hence, it is likely that BER is activated by DNA damage in PD instead of constitutively impaired (Fig. 2). However, a clear conclusion has still not been reached. The trends of BER proteins in PD are also divergent. For example, XRCC1, a key mediator of the final gap sealing in BER, was found to be negatively associated with sporadic PD risk in women in an Italian cohort. [117], but the Arg399Gln polymorphism of XRCC1 was shown to be positively associated with the risk of PD [118,119]. Hence, how the overall activity of BER changes in PD is still an open question, and the only assertion thus far is that the function of BER in PD and AD is not identical (Table 1).

In NER, ERCC1 contributes to the cleavage of nucleotides, and PCNA participates in the synthesis of new DNA strands. Interestingly, it has been shown that ERCC1 mutant mouse defects in NER mimicked the phenotype of PD [120]. Attenuated expression of PCNA was detected in a PD cell model in which PC12 cells were treated with MPP^+^ [121]. These studies reinforced the importance of NER in PD. Nevertheless, whether NER is diminished in the PD brain is still unclear.

Next, DSB repair in PD brain will be discussed. ATM, which is the initiator of DSB repair as described above, was found to be phosphorylated in dopaminergic neurons from a synucleinopathic PD mouse model [16]. This phosphorylation is an indicator of the activity of ATM because ATM is known to exhibit autophosphorylation when activated. Moreover, transgenic mice deficient in ATM demonstrated an abatement of dopaminergic neurons, which is a representative symptom of PD [122]. These findings indicated that ATM-dependent DSB repair is possibly activated in the PD brain. Nevertheless, based on another mouse PD model that overexpresses the A53T mutant of human α-synuclein, the duration of γH2AX cleanup was elongated compared to that of the control, as an indicator of suppressed DNA repair activity [123]. Therefore, the overall trend of DSB efficacy remains an open question.

PARP1, which was found to be elevated in the AD brain, was also increased in the lateral substantia nigra of PD patients [124]. PARP1 deletion [125] or inhibition [126] showed protective effects in PD animal models. A recent study suggested that the activation of PARP1 directly results from the aggregation of α-synuclein in PD neurons [126]. These findings indicate that PARP1 activation might be a common event during both PD and AD. However, whether PARP1-dependent DNA repair is involved in these neurodegenerative diseases is still unclear. It cannot be ruled out that PARP1 plays its role through mechanisms other than DNA repair.

Interestingly, some proteins that are known to play key roles in PD have been shown to directly mediate DNA repair. For example, α-synuclein, which is known as a hallmark of PD, was found to directly bind with double-stranded DNA and facilitate NHEJ [127]. Moreover, Parkin, whose mutations were found in inherited PD, was also reported to have an association with DNA repair in a bioinformatics study [128]. Cellular biological studies also revealed that Parkin protects not only mtDNA [129] but also nuclear DNA, as it translocates to the nucleus upon oxidative stress [130] and facilitates NER via interaction with PCNA [131]. Hence, it is likely that mutations in α-synuclein or Parkin may induce DNA repair deficiency and subsequently increase genome instability. However, this still needs to be tested in PD patients or disease models.

In summary, although DNA damage was found in both PD and AD, the underlying mechanisms might be different. For example, the BER components OGG1 and Pol β are upregulated in PD but downregulated in AD. Hence, it could be hypothesized that DNA oxidation in the PD brain induces the activation of the BER pathway. Moreover, deficiency of NHEJ and NER might be involved in the pathogenesis of PD (Fig. 2 andTable 1), at least partly due to dysfunction of the key PD related proteins, α-synuclein and Parkin. Nonetheless, the current understanding of DNA repair in PD is still very limited.

### 4.3 Amyotrophic lateral sclerosis (ALS)

ALS is a fatal neurodegenerative disease that results from the loss of motor neurons in the spinal cord and motor cortex. ALS patients show dysfunctions in muscle control, leading to muscle atrophy and weakness, difficulty in movements, slurred speech, trouble swallowing and even respiratory failure. Like AD and PD, the majority of ALS is sporadic, and its pathology is also not fully understood. To our current understanding, dysfunction mutations in a crucial antioxidative enzyme, copper Cu/Zn-superoxide dismutase 1 (SOD1), are associated with ALS, indicating the involvement of oxidative stress. Moreover, two nucleic acid binding proteins, fused in sarcoma (FUS) [132] and TAR DNA binding protein 43 (TDP43) [133], have also been revealed as key pathogenic proteins of ALS [134].

The pathological role of DNA damage and repair in ALS has also been a concern for a long time. The earliest hypothesis that deficiency of DNA repair is a cause of ALS can be traced back to the early 1980s [135]. In the case of BER, crucial mediators, such as OGG1, XRCC1, and APE1, were studied in ALS. In mutated SOD1 transgenic ALS mice, the expression of the nuclear form but not the mitochondrial form of OGG1 was increased [136], suggesting the potential involvement of gDNA repair. In addition, the Arg399Gln polymorphisms in the XRCC1 gene [137] and the Ser326Cys polymorphism of OGG1 [70] both exhibited associations with sporadic ALS. From these findings, the involvement of the BER pathway in ALS can be speculated.

More studies focusing on another key BER protein, APE1, have been conducted. APE1 was found to be downregulated in both expression level and activity in the frontal cortex of ALS patients [138]. Using SOD1-mutated mice as an ALS model, APE1 was found to be decreased in spinal motor neurons in the early presymptomatic stage before significant neuronal death was detected [139,140]. In contrast, another study reported the enhancement of APE1 expression in both motor neurons and astrocytes in the spinal cord of ALS patients [141]. Although the trend of APE1 expression in ALS is still under debate, mutations in the APE gene have been identified in ALS patients [142]. Meanwhile, in the SOD1-mutated ALS model, ER stress-induced nuclear translocation of APE1 was impaired [143]. Hence, polymorphisms and mislocalization of APE1, rather than alterations in expression level, may be the cause of reduced BER activity in ALS (Fig. 2).

In addition to oxidative lesions, robust DSBs were also found in ALS [144,145]. The kinase activity [144,146] and phosphorylation level [147] of ATM were both enhanced in ALS motor neurons. Moreover, the expression levels of BRCA1 [146], PARP1 [147,148], and Ku80 [149] were all elevated. Interestingly, inhibition of overactivated Ku80 was found to be protective against neurodegeneration in ALS. Since Ku80 plays a key role in the NHEJ pathway, it is likely that DSB repair, especially NHEJ repair, is activated in ALS motor neurons.

However, inhibition of DSB repair in ALS has also been reported. It was found that a key protein in the etiology of ALS, FUS, can be recruited to the site of DSBs and interact with HDAC1, hence playing a direct role in the repair of DSBs. Moreover, an FUS mutant that harbors mutations of familial ALS exhibited deficiency of HDAC1 interaction and impaired DNA repair, suggesting reduced DNA repair capacity specifically in ALS with FUS mutations [150]. FUS is also a substrate of DNAPKcs and is activated by phosphorylation of DNAPKcs in NHEJ [151]. FUS also contributes to BER by activating Lig3 and translocating XRCC1/Lig3 to the DNA damage site to complete DNA nick ligation [152]. In addition to FUS, another important protein in ALS, TDP43, also participates in DNA repair involving transcription-associated DNA damage [153]. It was also reported that TDP-43 plays a role in NHEJ by recruiting the XRCC4/Lig4 complex at DSB sites [154]. The final step of NHEJ is blocked in TDP-43-mutated ALS patients [155,156]. Similar to PD, ALS-associated mutations in FUS or TDP-43 likely lead to inherent loss of some type of DNA repair function, at least NHEJ function, even if the abundance of functional Ku80 is elevated (Fig. 2).

Taken together, how the activity of DNA repair is affected in ALS is complicated (Table 1). Patients with FUS or TDP43 mutations might have defects in NHEJ and BER. The trend is still unclear but could be increasing, as ATM and BRAC2 were found to be upregulated. The role of DNA repair in ALS still requires further studies to be fully elucidated.

### 4.4 Huntington's disease (HD)

HD is another fatal neurodegenerative disease, although it is relatively rare. Patients with HD demonstrate abnormal movements such as involuntary jerking or writhing, cognitive impairment, and psychiatric disorders. In contrast to AD, PD and ALS, the majority of HD is familial. It is an autosomal dominant inherited disease due to CAG expansion in the Huntingtin (HTT) gene that produces polyglutamine mutant HTT (mHTT) protein. The expression of mHTT progressively induces neurodegeneration in the brain, especially in the striatum and cortex [157,158].

First, it needs to be noted that the expansion of CAG in the HD gene depends on the activity of MMR. In other words, the MMR pathway plays a positive role in the pathogenesis of HA. Key components of the MMR pathway, including MLH1 [159,160], MLH3 [160], MSH2 [161] and MSH3 [162], were found to participate in the etiology of HD. In this special case, DNA repair does not act as a protector of the genome but an inducer of mutations. Hence, it will not be discussed in detail here.

DNA damage, such as DSBs, was also found in HD [163]. In addition, the repair mechanisms have also been studied. Regarding ATM, the key kinase that mediates the repair of DSBs, elevation of its activity was observed in brain tissue from HD patients or model mice [164]. Consistent with this result, the activation of ATM was also found in cells derived from HD mice [164], in PC12 cells expressing mHTT [163], and in a Tet-Off PC12 cell line mimicking HD [165]. The increased activation of ATM suggests the elevation of DSB repair capacity. However, in another study using HD fibroblasts, ATM-dependent DSB repair activity was impaired, as the kinetics of recovery from irradiation-induced DNA damage were found to be slower [166].

A plausible explanation for this discrepancy is that some downstream proteins of ATM in the DNA repair pathway are blocked in HD. In addition, a possible candidate here is HTT itself. Studies using fibroblasts from HD patients revealed that HTT directly participates in DNA repair by translocating to the damage site and recruiting other DNA repair proteins as a scaffold, which requires the kinase activity of ATM [167]. Hence, pathogenic mutations in HTT possibly eliminate the repair function of HTT and thereby block the ATM-triggered pathway. In this case, ATM fails to rescue destruction of the genome and is even overactivated. The overactivation of ATM might lead to more severe injury to the cell through alternative mechanisms other than DNA repair. It was reported that inhibition of ATM activity ameliorates injury in both transgenic mouse models and cell models [164]. In summary, the activity of ATM increases during HD, leading to exacerbation of cell damage without facilitation of DNA repair.

In addition, HTT also mediates TC-NER and NHEJ. Mutant HTT destroys the functional TC-NER complex by impairing the activity of PNKP and ATXN3 and subsequently suppresses DNA repair in HD mice and cell models [168]. Moreover, mutated HTT was found to impair NHEJ by disrupting the formation of the Ku70/Ku80 heterodimer in striatal neurons from transgenic HD mice [169]. All these findings consistently showed decreased DNA repair activity in HD (Figure 2).

## 5. Conclusions and perspectives

In summary, extensive DNA damage has been observed in various neurodegenerative diseases. However, whether dysfunction of DNA repair is a primary etiology of neurodegenerative diseases has remained unclear for decades. Many studies have attempted to determine whether magnified injury in the genome is associated with enhanced or reduced activity of various DNA repair pathways. It has been found that different types of diseases, different DNA repair pathways, and even different brain regions show distinct results. In brief, based on the current findings summarized above, BER is likely downregulated in AD and ALS but upregulated in PD, while NHEJ is likely impaired in all four types of neurodegenerative diseases discussed above. For HR, the trends are still to be determined, but the enhancement of ATM activity possibly indicates the increase of HR activity in PD, ALS, and HD (Fig. 2). Notably, in most cases, discrepancies still exist, and further studies are required in the future. However, at best, it can be concluded that the role of DNA repair in different neurodegenerative diseases is not identical, although similar DNA damage is observed.

Interestingly, downregulation of both BER and NHEJ pathways was observed in AD (Fig. 2). This is consistent with our proposed mechanism 1 inFig. 1. Hence, the impairment of the repair of DNA damage, including both oxidative bases and DSBs, is likely one of the primary causes of AD. Therapies and drugs targeting these pathways may potentially help the treatment of AD. For PD, it is more likely that the BER pathway is not involved in the pathology, as an increase in BER activity has been found. Meanwhile, a downregulation of NHEJ was also observed, indicating the possible involvement of DSB repair (Fig. 2). Regarding this, the NHEJ, but not BER, pathway might include potential drug target for PD. To date, data for ALS and HD are still limited, but ALS is somehow similar to AD in that both BER and NHEJ are likely involved according to current findings (Fig. 2).

A flaw of the studies summarized above is that the true DNA repair efficiency was not directly measured. In most studies, the abundance of mRNA or protein of key genes or the enzymatic activity of key proteins was utilized to estimate the efficiency of the corresponding DNA repair pathway. However, these measurements are not equal to the DNA repair activity. Indeed, the activity of a whole pathway could be suppressed if a downstream enzyme is inhibited, even if one upstream protein is activated. For example, ATM is usually activated in PD, whereas downstream ATM can be blocked by aggregation of α-synuclein, resulting in defects in DSB repair. In this case, no final conclusions can be made simply based on the measurement of each single protein. Some studies evaluated the activity of repair by quantification of the kinetics of the cleanup of DNA damage markers following external stress. Such measurement is indirect, although it can provide important information. Theoretically, delayed ROS scavenging might also retard the recovery of genome destruction as well as deficiency of DNA repair. To address this problem, the DNA repair efficiency in degenerative brains needs to be measured directly in future studies.

Furthermore, changes in DNA repair proteins may play additional roles in neurodegeneration mechanisms other than DNA repair. Again, ATM can be used as an example. ATM is a multifunctional protein, and overactivation of ATM might stimulate a series of events via phosphorylation of numerous substrates. If one of its downstream pathways is blocked, such as DSB repair in PD, other downstream mechanisms might be activated and contribute to the etiology. Hence, alternative functions of these proteins should receive more attention in future studies.

Finally, the role of DNA repair in neurodegenerative diseases is complex, and current understanding is still in a preliminary stage. To combat these age-related diseases, further studies focusing on DNA repair mechanisms in degenerative brains will provide crucial pieces of the puzzle for understanding the pathologies underlying neurodegenerative diseases.
